# Dosage strength is associated with medication persistence with Ginkgo biloba drug products: a cohort study of ambulatory drug claims data in Germany

**DOI:** 10.1186/1472-6882-13-278

**Published:** 2013-10-24

**Authors:** Sittah Czeche, Katrin Schüssel, Alexandra Franzmann, Martin Burkart, Martin Schulz

**Affiliations:** 1 German Institute for Drug Use Evaluation (DAPI), Jägerstrasse 49/50, 10117 Berlin, Germany; 2 Dr. Willmar Schwabe GmbH & Co. KG Pharmaceuticals, Willmar-Schwabe-Str. 4, 76227 Karlsruhe, Germany

**Keywords:** Ginkgo biloba, Adherence, Persistence, Prescription, Drug claims database, Cohort study

## Abstract

**Background:**

*Ginkgo biloba* drugs (Gb) are reimbursed within the German statutory health insurance (SHI) scheme for treatment of dementia. In 2008, a novel Gb product containing 240 mg Ginkgo extract EGb761^®^ per tablet was introduced aiming to facilitate medication use by incorporating the recommended daily dose in one single tablet. The aim of this study was to evaluate the relationship between dosage strength and persistence in a representative population of patients treated with Gb.

**Methods:**

Retrospective cohort study in ambulatory drug claims database within the German SHI system. Persistence was defined as continuous treatment with an allowable gap of 20% between refills. Multivariate regression models were conducted to identify variables associated with persistence.

**Results:**

Among 13,810 patients initiating treatment with Gb in 2008, 430 (3.1%) received a dosage strength of 240 mg, 7,070 (51.2%) a dosage strength of 120 mg and 6,310 (45.7%) dosage strengths containing less than 120 mg Gb per tablet. After 6 months, persistence was highest for patients treated with the 240 mg dosage form (22.8% of patients), although persistence was low in general (5.7% and 0% of patients treated with 120 mg and less than 120 mg, respectively). Risk for non-persistence was reduced in patients receiving 240 mg products compared to 120 mg (HR = 0.63; 95%CI 0.57 – 0.70).

**Conclusions:**

Patients initially treated with Gb 240 mg were more persistent compared to those receiving lower dosage strengths. Nevertheless, persistence with Gb therapy is generally low and should be improved in order to better realize therapeutic effects.

## Background

Dementias are diseases of old age and will gain importance with increasing life expectancy. Today, 1.3 million people with dementia live in Germany with an incidence of about 200,000 per year [1]. It is assumed that dementia results in a major burden to health care systems and society as a whole [2]. So far, there is no cure for dementias but several compounds have been approved for symptomatic treatment to improve cognitive abilities, other mental functions, and activities of daily living: cholinesterase inhibitors (ChEI), the non-competitive NMDA receptor antagonist memantine and standardized *Ginkgo biloba* dry leaf extracts (Gb) [3].

In Germany, Gb is approved as a non-prescription drug for the symptomatic treatment of mental losses due to organic brain syndrome within the framework of a general therapeutic concept in case of progressive impairment or loss of mental capacities (dementia syndrome), for vertigo, for tinnitus and for peripheral arterial occlusive disease. However, only the treatment of dementia is a reimbursable indication within the German statutory health insurance (SHI) system. The exact mechanisms of action of Gb have not been established so far, but effects of Gb constituents on mitochondrial function are being discussed [4,5]. From clinical trials with Gb, it has been estimated that Gb delays progression of symptoms in Alzheimer’s dementia by about 6–9 months [6]. In 2008, the German Institute for Quality and Efficiency in Health Care (IQWiG) published a favorable report on the benefit of Gb in mild-to-moderate Alzheimer’s disease for the therapeutic goal 'activities of daily living’ [7]. Moreover, an indication of a benefit of Gb regarding 'cognitive function’ , 'general psychopathological symptoms’ , and 'quality of life of caregivers’ was found. However, the recommendations were restricted to a daily dose of 240 mg and to the ginkgo biloba special extract EGb 761^®^. For lower daily doses, there was no conclusive evidence of a benefit.

A recent study among primary care physicians specialized in complementary and alternative medicine in Germany found that prescribing of Gb was strongly associated with diagnosis of Alzheimer’s disease versus other types of dementia [8]. Moreover, in this specialized group of physicians, Gb was the most frequent drug chosen for treatment of dementia in more than two thirds of patients [8]. However, prescribing rates of Gb in the general SHI population in Germany are much lower with approximately only 14% of antidementia drug prescriptions or 5,4% of DDDs constituting Gb drugs [9,10].

It is apparent that the duration of therapy, the continuous supply of the patient with medication as well as the appropriate taking of medication are a prerequisite for efficacy of any drug and thus, for a successful therapy [11]. For drugs to become effective in dementia, treatment duration should be sufficiently long: the European Medicines Agency (EMA) recommends trials of at least 24 weeks to assess a therapeutic effect in dementia for attainment of drug approval [12], and the IQWiG evaluated only trials of a duration of at least 16 weeks for assessment of effectiveness [7]. In placebo-controlled trials with EGb 761^®^, therapeutic effects were more pronounced after 24 weeks than after 12 weeks of treatment [13,14].

Non-adherence is a major source of treatment failure [15]. Non-adherence reduces the chance to experience a treatment benefit, and non-appearance of an expected treatment effect can result in premature discontinuation. Reducing the complexity of medication regimens has been found to be one of the most effective measures to improve adherence [16,17]. This holds especially true in patients with memory disorders [18].

In 2008, Gb products for the treatment of dementia were available at strengths of 30, 40, 50, 60, 80, or 120 mg per tablet and as drops. To achieve the recommended daily dose of 240 mg, two to eight single doses would have been required. Therefore, a novel product containing 240 mg EGb 761^®^ per tablet was developed and introduced in 2008 with the aim of facilitating medication use by containing the recommended daily dose in one single tablet.

The aim of this study was to evaluate the relationship between dosage strength and treatment duration (persistence) in a representative population of patients treated with Gb. We hypothesized that persistence is higher with the 240 mg dosage form.

## Methods

### DAPI database

The study was designed as a retrospective cohort study in the DAPI database (http://www.dapi.de), which comprises claims data of prescribed drugs dispensed by community pharmacies in Germany at the expense of the SHI funds. For each prescription dispensed, the collected data include a de-identified patient code, the date of prescription, the product identifier (PZN) and the quantity of drug product dispensed. Via the PZN linkage to drug information systems is possible, allowing for characterization of the product with regard to e.g. the amount and type of active ingredient, dosage form, package size etc. Almost 90% of the German population is covered by the SHI system [19]. The DAPI database contains a representative sample of over 80% of the community pharmacies throughout Germany. Information about self-medication, drugs at the expense of private health insurance companies, physician samples and drugs dispensed during periods of hospitalization is not available.

The database is not publicly accessible, however studies utilizing data from the DAPI database can be performed on request - subject to the condition that the purpose of the study complies with the DAPI’s statutes. The DAPI is a non-profit association aiming at the advancement of scientific research in drug utilization and drug safety.

Ethical approval was not required for the present study, as (i) the data are routinely collected during the process of care without an intervention and (ii) the data comprise de-identified drug claims data being devoid of any personal information, thus precluding re-identification of patients. The database and anonymisation procedures were approved by the responsible data protection authority, at the time of study the regional administrative authority of Darmstadt in Hesse, in compliance with German legislation on data privacy and utilization of data from drug claims.

### Study design

A cohort study was performed comparing three treatment groups according to the dosage strength of the first Gb prescription (index prescription) between January 1st and December 31st 2008:

• patients with an index prescription of 240 mg Gb dosage strength

• patients with an index prescription of 120 mg Gb dosage strength

• patients with an index prescription with a dosage strength of less than 120 mg.

Furthermore, patients had to fulfill the following inclusion criteria:

• incident Gb therapy (i.e. no prescription of Gb during 365 days before the index date),

• registration in the database 730 to 365 days prior to index date,

• unambiguous assignment to a study group at the index date.

Medical information on the exact indication or co-morbidities as well as demographic information is not available in the database. However, reimbursement restrictions within the SHI scheme allow reimbursement of Gb only for dementia. Therefore, it can be assumed that Gb was prescribed for this indication and not for other labeled indications (such as tinnitus etc.). In addition, the following covariates have been retrieved from the database for further characterization of patients and co-morbidities, and these were also considered as potential risk factors associated with non-persistence:

• medical specialty of initial prescriber: general practitioners, neurologists, internal medicine specialists and other specialties were considered, as prescribing behavior may depend on medical specialty,

• region of the prescriber: western and eastern Germany, as there might be differences in prescribing Gb, because there is a forty years’ tradition to prescribe Gb in the former Federal Republic of Germany (west), but not in the former German Democratic Republic (east),

• insurance membership status at index date: mandatory member, family member or retired person, as this variable is a proxy for the demographic variable age in the database,

• pre-treatment within the 180 days before the index date with

• further antidementia substances (Anatomical Therapeutic Chemical (ATC)-code N06D excluding Gb),

• antidepressants (ATC-code N06A),

• nootropics (ATC-code N06BX),

• or drugs indicating the possibility of a co-existing disease for which Gb may be used but not reimbursed: for indication tinnitus: pentoxifylline (ATC-code C04AD03), for indication peripheral arterial occlusive disease cilostazol (ATC-code B01AC23) and naftidrofuryl (ATC-code C04AX21), and antivertigo preparations containing betahistine, flunarizine or cinnarizine (ATC-code N07CA).

• Number of different ATC 3rd level codes per patient prescribed within the 180 days prior to index date except those ATC 3rd level codes mentioned above, as the number of different ATC 3rd level codes is supposed to indicate indirectly the degree of co-morbidity.

Patients were excluded if the medical specialty of the prescribed physician, the region or insurance membership status of the index prescription were not available or defined ambiguously.

### Outcome

Persistence was determined as the primary study outcome during 365 days following treatment initiation. It was defined as continuous treatment assuming that any Gb medication prescribed is completely taken at the recommended dose of 240 mg per day, with temporal lapses between subsequent prescriptions not exceeding an allowable gap of 20% of the duration of the previous medication supply [20]. Stockpiling, i.e. medication oversupply due to early refill of a prescription, was taken into account in the calculation of persistence. Any switch to another Gb medication (different drug product and/or different dosage strength) after the index prescription was permitted and was not regarded as treatment discontinuation. If a patient had not refilled a prescription within a predetermined number of days after running out of medication supply plus the allowable gap of 20%, the patient was considered to have discontinued therapy and therefore was classified as non-persistent at the end of the allowable gap [21]. Patients were censored if they were lost to follow-up (i.e. recording of the last prescription of any drug in the database) or at the end of the study period (12 months after the index date). A change of the prescribing physician did not result in loss to follow-up.

The persistence definition requires assumptions about the prescribed daily dose (i.e. 240 mg). However, the prescribed daily dose is not available in the reimbursement database and may not apply to all patients, e.g. if prescribers deliberately chose a lower dose for an individual patient. Therefore, two additional measures of treatment continuation were employed, which do not depend on a dosage assumption. First of all, the length of therapy was used to calculate the proportion of days covered with Gb medication from the index date to the date of the last Gb prescription within the study period (12 months) assuming that the patient had been dispensed enough medication to cover the interjacent period. Patients without a refill prescription were by definition excluded from this analysis. Secondly, the number of patients with at least one subsequent refill prescription within 12 months after the index prescription was determined.

Furthermore, we investigated the proportion of patients changing their study group, i.e. who switched between Gb products of different dosage strengths.

### Statistical analyses

For persistence, length of therapy and refill prescriptions, descriptive statistics (i.e. proportions and median values) were determined. A log-rank test was used to compare persistence between the three study cohorts and Cox proportional hazard models were conducted to identify and control for factors associated with non-persistence. For the analyses of the proportion of patients with a refill prescription, logistic regression models were used. Significance levels were set at α = 0.05.

All statistical analyses were performed using SPSS for Windows version 14 and R version 2.13.0.

## Results

### Cohort attrition

**Figure 1:**
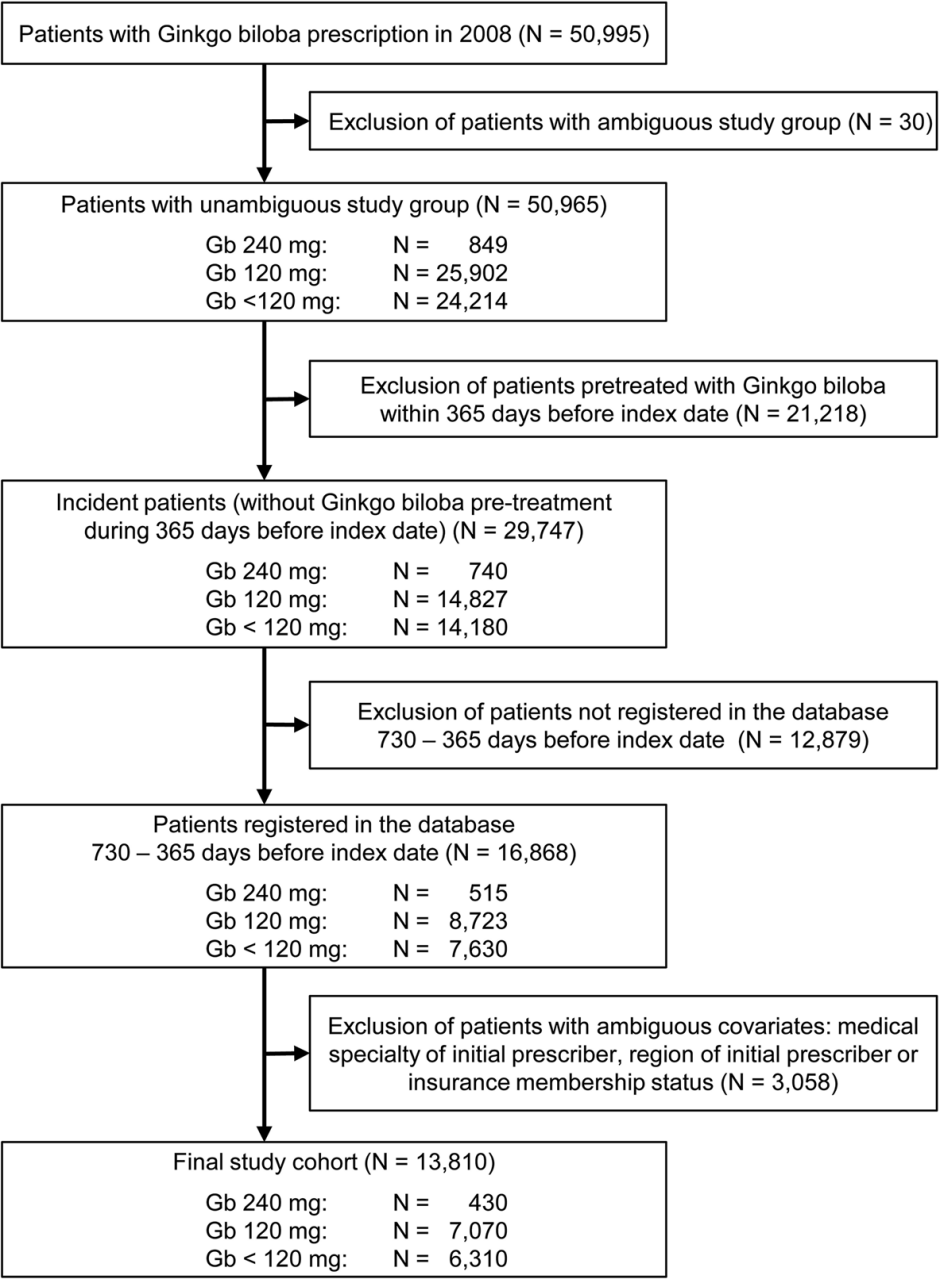
Inclusion and exclusion criteria of the study cohort.

In the DAPI database, 50,995 patients were identified who received at least one prescription of Gb during the index period between January 1st and December 31st 2008. Pre-treatment with Gb during 365 days prior to index date was the main reason for exclusion of the majority of patients (i.e. 41.6%) from the cohort. Nearly a quarter of patients (25.3%) lacked availability in the database in the period of 730 – 365 days before index date. After implementation of all inclusion and exclusion criteria, 13,810 patients remained in the final study cohort, of whom 430 patients (3.1%) were treated with Gb at a strength of 240 mg, 7,070 patients (51.2%) at a strength of 120 mg and 6,310 patients (45.7%) at strengths of less than 120 mg (i.e. 30, 40, 50, 60 or 80 mg) at the date of the index prescription (see Figure 1).

### Characterization of the final study population

The baseline characteristics of the study groups are detailed in Table 1. As Gb with a strength of 240 mg in one tablet had just been introduced on the German market in 2008 [22], the lowest proportion of patients (430 patients or 3.1% of total study cohort) received prescriptions with this strength, while a similar number of patients were prescribed strengths of 120 mg or less than 120 mg (7,070 and 6,310 patients or 51.2% and 45.7% of total study cohort, respectively). Generally, Gb medication has mainly been prescribed by general practitioners. However, the proportion of patients receiving prescriptions of 240 mg or 120 mg Gb strength was higher in the group of neurologists (32.3% and 28.8% respectively), while physicians of other specialties tended to more frequently issue prescriptions for less than 120 mg Gb strength. The majority of all patients receiving Gb were retired (74.3% – 82.1%). Within the pre-treatment period of 180 days before the index date, only few patients received co-medication with drugs for indications possibly related to Gb use (i.e. antidementia agents, nootropics, drugs used for the treatment of peripheral arterial occlusive disease, vertigo or tinnitus). For example, antidementia agents were used in 8.0% to 12.6% of patients. However, pre-treatment with antidepressants was quite common (38.0% – 44.4%). Polypharmacy, defined as prescriptions of 5 or more different ATC 3rd level drugs within 180 days before the index date, was present in approximately 80% of the patients.

**Table 1:** Baseline characteristics of the study cohorts

	**Number of patients (% of study group)**		
	**240 mg dosage strength**	**120 mg dosage strength**	**< 120 mg dosage strength**
Number of patients	430	7,070	6,310
Medical specialty of initial prescriber			
General practitioner	182 (42.3)	3,230 (45.7)	2,978 (47.2)
Neurologist	139 (32.3)	2,038 (28.8)	887 (14.1)
Internist	56 (13.0)	950 (13.4)	896 (14.2)
Other	53 (12.3)	852 (12.1)	1,549 (24.5)
Region of the initial prescriber			
Western Germany	299 (69.5)	5,499 (77.8)	5,500 (87.2)
Eastern Germany	131 (30.5)	1,571 (22.2)	810 (12.8)
Insurance membership status			
Retired	353 (82.1)	5,814 (82.2)	4,687 (74.3)
Mandatory member	62 (14.4)	1,029 (14.6)	994 (15.8)
Family member	15 (3.5)	227 (3.2)	629 (10.0)
Pre-treatment 180 days prior to index date with			
Antidepressants	181 (42.1)	3,136 (44.4)	2,395 (38.0)
Antidementia drugs	54 (12.6)	782 (11.1)	505 (8.0)
Nootropics	24 (5.6)	379 (5.4)	236 (3.7)
Antivertigo preparations (betahistine/flunarizine/cinnarizine)	50 (11.6)	720 (10.2)	639 (10.1)
Pentoxifylline	16 (3.7)	372 (5.3)	366 (5.8)
Drugs for treatment of peripheral arterial occlusive disease (cilostazol/naftidrofuryl)	16 (3.7)	189 (2.7)	181 (2.9)
≥ 5 different ATC third level drugs within 180 days prior to index date	357 (83.0)	5,850 (82.7)	5,023 (79.6)
Patients with follow-up prescription	227 (52.8)	3,202 (45.3)	2,154 (34.1)

### Results of the follow-up period of 365 days

**Figure 2:**
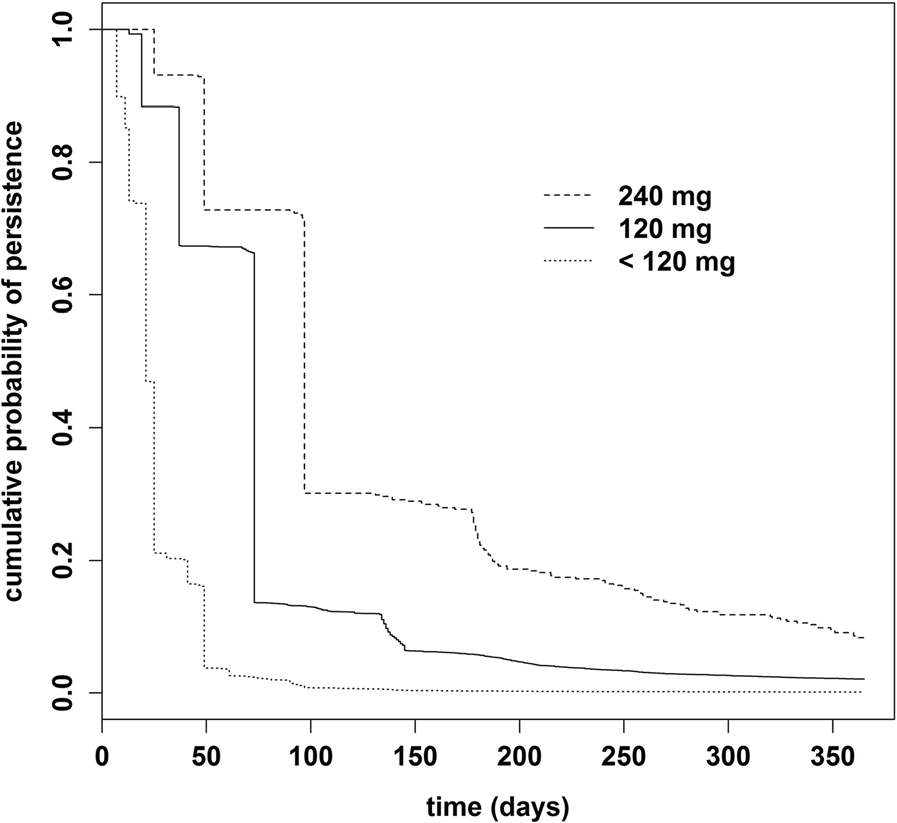
Kaplan-Meier curves showing the cumulative probabilities of continuation of therapy (persistence) in patients treated at index prescription with Gb at 240 mg dosage strength, 120 mg dosage strength, or less than 120 mg dosage strength.

In the study group treated with 240 mg Gb at the index date, the proportion of persistent patients was higher compared to the other study groups. Kaplan-Meier curves of treatment persistence with Gb medication until discontinuation of therapy are shown in Figure 2. Differences between the study groups were statistically significant (log-rank test, p < 0.0001). The staircase pattern of the curves results from the different package sizes that were dispensed. Median duration of persistence was 97, 73 or 21 days for patients treated initially with 240 mg, 120 mg, or less than 120 mg Gb dosage strength, respectively. After 6 months, cumulative probability of persistence was 22.8% for patients treated with 240 mg Gb dosage strength, 5.7% for 120 mg dosage strength and already close to 0% for patients treated with less than 120 mg Gb dosage strength. After 12 months, only 8.4% of patients with 240 mg Gb and 2.1% with 120 mg Gb were still persistent.

In the unadjusted Cox regression model the risk for being non-persistent was decreased by 37% for patients treated with 240 mg Gb dosage strength vs. 120 mg (HR 0.63; 95% CI: 0.57 – 0.70), whereas the risk for non-persistence was increased by 4.5 times for patients treated with less than 120 mg dosage strength vs. 120 mg (HR 4.54; 95% CI: 4.36 – 4.73). In the adjusted multivariate Cox regression model, further covariates were associated with a reduced risk for non-persistence, e.g. neurologist as medical specialty of prescribing physician at index date, eastern Germany as region of the prescriber or pre-treatment with antidementia drugs (see Table 2). Conversely, the hazard for non-persistence was increased for patients with insurance status member or family member (relative to retired persons).

**Table 2:** Adjusted hazard ratios (HR) for non-persistence and 95% confidence intervals (CI) in the Cox regression model

	**Adjusted HR**	**95% CI**
Study group (ref. 120 mg Gb dosage strength)		
240 mg Gb dosage strength	0.64 *	0.57-0.71
< 120 mg Gb dosage strength	4.36 *	4.18-4.54
Medical specialty of initial prescriber (ref.: general practitioner)		
Neurologist	0.93 *	0.89-0.97
Internist	0.96	0.91-1.01
Other	1.43 *	1.36-1.50
Region of initial prescriber (ref.: Western Germany)		
Eastern Germany	0.74 *	0.71-0.77
Insurance membership status (ref.: retired)		
Mandatory member	1.23 *	1.17-1.29
Family member	1.61 *	1.50-1.73
Pre-treatment 180 days prior to index date (ref.: none)		
Antidepressants	0.97	0.93-1.01
Antidementia drugs	0.86 *	0.81-0.91
Nootropics	0.92	0.85-1.00
Antivertigo preparations (betahistine/flunarizine/cinnarizine)	0.99	0.94-1.05
Pentoxifylline	1.07	0.99-1.15
Drugs for treatment of peripheral arterial occlusive disease (cilostazol/naftidrofuryl)	0.99	0.89-1.10
Number of different ATC third level drugs prescribed within 180 days prior to index date	1.00	1.00-1.00

* HR significant at p < 0.05.

As the persistence method depends on the assumption of a prescribed daily dose of 240 mg, further dose-independent measures of treatment continuation were employed. The length of therapy was calculated as the time between the first and last Gb prescription during the observation period of 12 months after the index date, assuming that the patient had been dispensed enough medication to cover the interjacent time period. For this sensitivity analysis, only patients with at least one refill prescription were included (5,583 patients out of 13,810 in the total study cohort). The median of the length of therapy was highest with 281 days in the 240 mg Gb group followed by 250 days in the 120 mg group and 225 days in the group of patients treated with Gb dosage strengths of less than 120 mg, confirming the results from the persistence analysis. After 6 months, 68.2% of the 240 mg Gb patients, 63.4% of the 120 mg Gb patients and 57.9% of the patients treated with less than 120 mg dosage strengths were considered to be supplied with Gb medication.

As a further dose-independent method we determined the proportion of patients receiving at least one refill prescription after the index date. Again, the proportion of patients refilling at least one prescription was highest (52.8%) in the group of patients initially treated with 240 mg strength compared to 45.3% of patients treated with 120 mg and 34.1% of patients treated with less than 120 mg dosage strength. For logistic regression analysis, only patients with continuous follow-up in the DAPI database for at least 12 months after index date were included (12,191 patients out of 13,810 in the total study cohort). The unadjusted odds for receiving a refill prescription was increased for Gb 240 mg patients by 29% (OR 1.29; 95% CI: 1.05 – 1.59) and decreased for less than 120 mg Gb patients by 37% (OR 0.63; 95% CI: 0.58 – 0.68) relative to the group of patients treated with 120 mg Gb dosage strength. A similar association was found in the adjusted model: The odds to obtain a refill prescription was increased by 28% for 240 mg Gb patients (OR 1.28; 95% CI: 1.03 – 1.58) whereas it was decreased by 23% for less than 120 mg Gb (OR 0.77; 95% CI: 0.71 – 0.83) relative to patients treated with 120 mg Gb dosage strength.

Similar to the associations observed in the Cox regression analysis of persistence, the covariates internal medicine specialist and neurologist, eastern Germany as region of the prescribing physician as well as pre-treatment with antidementia drugs were associated with increased odds for a follow-up prescription, while insurance status member and family member were associated with reduced odds for a follow-up prescription relative to retired persons. Further covariates with a positive association of receiving a refill prescription were pre-treatment with antidepressants (OR 1.11; 95% CI: 1.03 – 1.26) and nootropics (OR 1.46; 95% CI: 1.23 – 1.73) in the logistic regression model.

However, the results from both regression models are limited by a lack of goodness-of-fit (i.e. the c-statistic for the logistic regression model was only 0.647) and in case of the Cox regression models by violations of the proportional hazard assumption.

Finally, switching the dosage strength during follow-up occurred only rarely: in the 240 mg Gb and less than 120 mg Gb groups, the proportions of patients switching the dosage strength were similar (13.2% and 12.9%, respectively), whereas in the 120 mg Gb group merely 7.3% of patients switched the dosage strength.

## Discussion

In the present study, a large cohort of 13,810 patients was identified from the DAPI database with a first prescription for Gb medication in 2008. Continuation of drug therapy within 12 months from treatment initiation was generally lower than expected in this cohort, although it was significantly higher in patients initiating therapy with the 240 mg dosage strength. After 6 months, the cumulative probability of persistence of patients in the 240 mg, 120 mg, or less than 120 mg groups decreased to 57.4%, 22.8%, or nearly 0%, respectively, and after 12 months to 8.3% in the 240 mg Gb group and to 2.1% in the 120 mg Gb group. Likewise, only a low proportion of patients filled a second prescription after Gb treatment initiation: 52.8% in the 240 mg dosage strength group, 45.3% in the 120 mg group and 34.1% in the less than 120 mg group. In analogy to the measures of persistence and refill prescriptions, the length of therapy was highest for patients treated with 240 mg dosage strength compared to study groups with lower dosage strengths.

These results imply that Gb drugs containing the recommended daily dose of 240 mg in a single tablet might be favorable for patients’ persistence - although according to the current label the tablet should be split and one half taken twice a day [22], i.e. the dosage interval thus remains unaltered compared to twice daily dosing of Gb products with 120 mg dosage strength. Nevertheless, we cannot exclude that patients may use the 240 mg product once daily, as this may be more practicable in real-life settings for patients with cognitive impairment and their care-givers [23]. A simpler and less frequent doses regime may increase patients’ adherence [16,17]. The efficacy and safety of a once daily regimen of 240 mg EGb 761^®^ has been demonstrated in several major placebo-controlled trials [13,14]. Hence, presumed once-daily dosing of the 240 mg Gb products - albeit contrary to the dosage instructions from the package insert - may in part explain the higher adherence (persistence) observed in the present study.

Noting that treatment duration of dementia should be at least 12 weeks [7,24] the persistence after 3 months may be sufficient only for the 240 mg Gb cohort of patients, whereas it is most probably inadequate for the other study groups. However, we cannot exclude that persistence in real-life is better than calculated from SHI reimbursement data, where data on self-medication are unavailable – especially since Gb is available without prescription and the largest share of Gb drugs in Germany is purchased via self-medication [25].

The low persistence levels observed in our study may also be explained by discontinuation of the drug due to side effects. However, serious side effects of Gb that could result in discontinuation of therapy were not observed in previous studies. Results of a meta-analysis have shown that the rates of side effects and discontinuation were not different between EGb 761^®^ and placebo [26]. The dropout rates in clinical trials of Gb have been variable, ranging from 1% to 62% [26], which may indicate that other factors, e.g. age, co-morbidities, personality traits [27] or unrealistic expectations on treatment effects may be associated with or explain discontinuation of Gb treatment.

Direct comparisons of persistence results from this study with other investigations are difficult, because, to the best of our knowledge, other data from large population-based non-interventional cohort studies with Gb treatment are not available. Nevertheless, persistence of patients with dementia was evaluated for ChEI or memantine, albeit with differences in the definition of persistence, inclusion and exclusion criteria or other operational definitions. A recent study based on SHI data in Germany reported that patients with dementia only rarely received continuous prescriptions with an appropriate dosage in each of four quarter years after treatment initiation: 44% of patients treated with ChEIs and 15% of patients treated with memantine [9]. Studies from other countries reported on persistence rates at 12 months of 33.6% for the ChEI donepezil and rivastigmine in Canada [28] and 45.3% for the ChEI rivastigmine and galantamine in France [29]. Persistence results from these studies with an allowable gap of 60 days between refills are higher compared to the Gb persistence rates in the present study where a gap of only 20% was allowed. As the 20% allowable gap is in all cases considerably lower than 60 days (e. g. for a prescription of 120 tablets with the longest coverage, the 20% allowable gap are 44 days after depletion of the package), these differences in methodology readily explain the lower persistence observed in our study. In the present study a relatively small gap was chosen, because Gb should be taken continuously with at the utmost only small gaps for its efficacy. Furthermore, instead of an absolute number of days as allowable gap, a relative gap of 20% was chosen in order to allow for the variable package sizes available on the market.

In the pre-treatment period, a large proportion of patients of all 3 study groups frequently received antidepressants. Findings from various studies have shown that there is evidence for an association between late-life depression and dementia, especially Alzheimer’s disease (AD) [30-32]. Still it is not clear whether a depression history is a risk factor for AD or whether neuropathologic changes in AD may result in a depression. However, the close relationship between both diseases could be a possible explanation that nearly half of patients across all three Gb treatment groups received antidepressants in the present study. Therapy with antidepressants was not associated with persistence and showed only a weak, but positive association with the odds of filling a refill prescription. In conclusion, the use of antidepressants in the present study does not seem to have a negative effect on persistence with Gb therapy.

The proportion of patients with pre-treatment with any antidementia drug was approximately equal, but low, in all study groups. Thus, it appears that patients in the present cohort suffer from mild to moderate symptoms of dementia, where Gb may be chosen as first treatment option before medication with ChEI or memantine was initiated. Alternatively, other antidementia drugs may not have been considered suitable for patients due to contraindications, interactions, side effects, or other than labeled dementia diagnoses. Although ChEI are among the recommended first-line drugs in the treatment of mild to moderate stages of AD [33], their use is limited due to side effects such as gastrointestinal disorders, dizziness, anorexia, or tremor [34]. For the group of patients pretreated with antidementia drugs, augmentation of therapy with Gb might have been pursued by the prescriber. There is evidence that a combination of both, ChEI and Gb, may be superior to a monotherapy with only ChEI or Gb and will be associated with efficacy and less side effects, as shown in an exploratory trial with donepezil and EGb 761^®^[35].

The present study has several limitations which should be taken into account for interpretation of results. Firstly, because in the database no information about drugs dispensed by community pharmacies without prescription (self-medication) or prescriptions at the expense of private health insurance companies is available, patients may be incorrectly interpreted as non-persistent, as already stated above. This also implies that the observed differences in persistence between different dosage strengths of Gb in this study may be biased if Gb medication acquisition via self-medication is non-differential for the different dosage strengths – although there is neither data available to support or refute this hypothesis. Moreover, patients may have incorrectly been classified as non-persistent due to periods of hospitalization or provision of free medication samples from their physician. Therefore, the persistence of patients taking Gb would be higher if all routes of medication supplies could have been captured. On the other hand, persistence may be overestimated from claims data, since it is assumed that any medication dispensed is completely taken by the patient, which is probably not the case.

Secondly, as in any observational and non-randomized study, the differences in persistence between the study groups treated with different Gb dosage strengths may be biased by unmeasured, and hence uncontrolled confounding. For example, a previous study found associations between age, personality factors, cognitive function and Gb adherence [27]. Such variables were not available in the present study. However, insurance membership status as a proxy variable for both socioeconomic status and age was included in the regressions models and an association with improved persistence for retired persons relative to other insurance membership categories was found, which confirms previous findings that socioeconomic factors or age may indeed affect Gb persistence. Nevertheless, we cannot exclude that confounder control with this proxy variable is still limited.

Furthermore, choice of Gb dosage strength may be related to physician factors such as prescribing preference, which in turn may be either directly or indirectly associated with different persistence with drug therapy. Little is known about physician prescribing preferences for dementia drugs [9] or more specifically for Gb [8]. Although a recent study reported on neurologists preferentially prescribing other antidementia drugs relative to Gb [8], data on prescribing preferences with regard to choosing the dosage strength of Gb are unavailable to the best of our knowledge. Also, direct evidence of an association between physician-related factors and persistence with dementia treatment is lacking, although an observational study reported that medication review by doctors was associated with better medication adherence in elderly patients [36]. The results from the present study suggest (i) that neurologists preferentially prescribe Gb with higher dosage strengths as well as (ii) that being treated by a neurologist is associated with better persistence, hence a potential confounding effect of physician-related factors on persistence cannot be ruled out. In summary, although available covariates such as specialty of prescribing physician were included in multivariate models in the present study, further important confounders may have been missed and associations between different Gb dosage strengths and persistence may still be biased by unmeasured confounding [37].

Thirdly, the persistence calculation required assumption of a prescribed daily dose and may not reflect the real daily dose for all patients. However, the dose-independent measures of therapy continuation led to essentially the same conclusions.

A strength of the current study is the size of the DAPI database covering more than 80% of community pharmacies throughout Germany. Hence, the sample of patients in the current study can be assumed to be representative for the SHI scheme. Moreover, refill persistence – as opposed to patient-reported measures – can be regarded as an objective measure of medication adherence [15].

## Conclusions

The results from this study provide evidence that treatment duration with Gb therapy is insufficient in a significant proportion of patients to receive the achievable benefit of therapy. Physicians and pharmacists alike should make efforts to stress the importance of adequate Gb persistence to patients, caregivers and relatives. Moreover, a dosage strength of 240 mg contained in a single dosage form – i.e. the recommended dosage for treatment of dementia per day – appears to be advantageous, especially for older patients with cognitive impairments who are often in need to take numerous other drugs. This observation deserves confirmation in prospective studies.

## Abbreviations

AD: Alzheimers’ disease; ATC code: Anatomical therapeutic chemical code; ChEI: Choline esterase inhibitors; CI: Confidence interval; DAPI: Deutsches Arzneiprüfungsinstitut e.V. (German Institute for Drug use Evaluation); EMA: European medicines agency; Gb: Ginkgo biloba; HR: Hazard ratio; IQWiG: Institut für Qualität und Wirtschaftlichkeit im Gesundheitswesen (Institute for Quality and Efficiency in Health Care); SHI: Statutory health insurance.

## Competing interests

This study was financially supported by and MB is an employee of Dr. Willmar Schwabe Pharmaceuticals, Karlsruhe, Germany, manufacturer of EGb 761^®^.

## Authors’ contributions

SC and KS designed the study, developed the study protocol, performed the statistical analyses and drafted the manuscript. AF approved the study protocol and contributed to the statistical analyses. MB initiated the study, contributed to the design and study protocol and critically revised the manuscript for important intellectual content. MS contributed to the study design and study protocol and made substantial contributions to the manuscript. All authors read and approved the final manuscript.

## Pre-publication history

The pre-publication history for this paper can be accessed here:

http://www.biomedcentral.com/1472-6882/13/278/prepub
